# Supplementary material for: The Burning Pain Transcriptome in the Mouse Primary Somatosensory Cortex
Source: Int J Mol Sci. 2025 Apr 9;26(8):3538. doi: 10.3390/ijms26083538 (PMC12027419; doi:10.3390/ijms26083538)
Supplement: Supplementary file 1 [file ijms-26-03538-s001.zip › ijms-3512996-supplementary.pdf]

The Burning Pain Transcriptome in the Mouse Primary Somatosensory Cortex

Virág Erdei[1,2a], *Zoltán Mészár*[1a] *and Angelika Varga*[1*]

[1]Department of Anatomy, Histology and Embryology, Faculty of Medicine, University of Debrecen, Debrecen, Hungary; meszarz@anat.med.unideb.hu

[2] Department of Radiology, Central Hospital of Northern Pest - Military Hospital, Budapest, Hungary; erdei.virag6@gmail.com

[*] Correspondence: varga.angelika@med.unideb.hu; Tel.: +36(52)255-567

[a] These authors contributed equally to this work.

Supplementary Table S1.

List of the top 15 upregulated and downregulated differentially expressed genes (DEGs) in the primary somatosensory cortex (S1) in response to burn injury (BI)

	Gene symbol	Gene name	Alteration in gene expression	Molecular function	Biological process	Fold-change *vs* control
1	**MT-ATP6**	Mitochondrially Encoded ATP Synthase 6	↑	proton-transporting ATP synthase activity	ATP biosynthetic process	2.69
2	**A4galt**	Alpha 1,4-Galactosyltransferase Synthase	↑	galactosyltransferase activity	lipid metabolic process<br>glycosphingolipid biosynthetic process	1.82
3	**ZFP566**	Zinc Finger Protein 566	↑	transcriptional regulator	negative regulation of transcription by RNA polymerase II	1.67
4	**CDR1**	Cerebellar Degeneration Related 1	↑	signal transduction protein	None by GO term	
5	**KIFC5B**	Kinesin Family Member C1	↑	microtubule binding activity and motor activity	microtubule-based movement	1.64
6	**LRG1**	Leucine Rich Alpha-2-Glycoprotein 1	↑	Protein binding	positive regulation of epithelial-to-mesenchymal transition<br>wound healing	1.56
7	**EGFL8**	EGF Like Domain Multiple 8	↑	signaling receptor binding	anatomical structure development	1.55
8	**RBM46**	RNA Binding Motif Protein 46	↑	nucleic acid binding	cell differentiation	1.54
9	**TMEM114**	Transmembrane Protein 114	↑	Protein binding	None by GO term	1.50
10	**NMB**	neuromedin B	↑	neuropeptide hormone activity	neuropeptide signaling pathway	1.49

11	**ESPL1**	Extra Spindle Pole Bodies Like 1, Separase	↑	cysteine-type peptidase activity	positive regulation of mitotic metaphase/anaphase transition	1.48
12	**GPR183**	G Protein-Coupled Receptor 183	↑	G protein-coupled receptor activity	G protein-coupled receptor signaling pathway<br>positive regulation of ERK1 and ERK2 cascade	1.48
13	**TAGLN**	Transgelin	↑	protein binding	epithelial cell differentiation and burn wound healing	1.46
14	**TNNC1**	Troponin C1, Slow Skeletal and Cardiac Type	↑	Protein binding	regulation of muscle filament sliding speed	1.45
15	**IQCD**	IQ Motif Containing D	↑	Protein binding<br>Component of the nexin-dynein regulatory complex	None by GO term	1.44
16	**BSN**	Bassoon Presynaptic Cytomatrix Protein	↓	presynaptic cytomatrix protein	presynaptic active zone assembly<br>chemical synaptic transmission	-2.25
17	**GRIK3**	Glutamate Ionotropic Receptor Kainate Type Subunit 3	↓	glutamate receptor 7	neuroinflammation and glutamatergic signaling<br>synaptic transmission	-2.26
18	**GPR161**	G Protein-Coupled Receptor 161	↓	G protein-coupled receptor activity	Key negative regulator of Shh signaling<br>G protein-coupled receptor signaling pathway	-2.26
19	**PSD3**	Pleckstrin And Sec7 Domain Containing 3	↓	Guanine nucleotide exchange factor for ARF6	regulation of ARF protein signal transduction	-2.27
20	**GRIN2A**	Glutamate Ionotropic Receptor NMDA Type Subunit 2A	↓	NMDA glutamate receptor activity	glutamate receptor signaling pathway<br>regulation of neuronal synaptic plasticity<br>sensory perception of pain	-2.34

21	**CBLN1**	cerebellin 1 Precursor	↓	protein binding	synapse integrity and synaptic plasticity	-2.38
22	**SNED1**	Sushi, Nidogen and EGF Like Domains 1	↓	protein binding	cell-matrix adhesion	-2.40
23	**PLXNA4**	Plexin A4	↓	Coreceptor for SEMA3A<br>Semaphoring receptor binding	negative regulation of cell adhesion<br>axon guidance	-2.41
24	**AGO2**	Argonaute RISC Catalytic Component 2	↓	nucleic acid binding	translational initiation	-2.43
25	**ADCY1**	Adenylate Cyclase Type I	↓	adenylate cyclase activity	cAMP biosynthetic process	-2.48
26	**KLF12**	KLF Transcription Factor 12	↓	DNA-binding transcription repressor activity	negative regulation of transcription by RNA polymerase II	-2.50
27	**Zdbf2**	Zinc Finger DBF-Type Containing 2	↓	nucleic acid binding	genomic imprinting	-2.52
28	**ERBB4**	Erb-B2 Receptor Tyrosine Kinase 4	↓	protein tyrosine kinase activity<br>transmembrane receptor protein tyrosine kinase activity	negative regulation of the apoptotic process<br>positive regulation of ERK1 and ERK2 cascade	-2.59
29	**SEMA5A**	Semaphorin 5A	↓	semaphorin receptor binding	negative regulation of cell adhesion<br>axon guidance	-2.83
30	**DCC**	DCC Netrin 1 Receptor	↓	netrin 1 receptor	axon guidance	-3.42

Supplementary Table S2.

List of the top 15 upregulated and downregulated differentially expressed genes (DEGs) in the primary somatosensory cortex (S1) in response to formalin-induced inflammatory pain (FA)

	Gene symbol	Gene name	Alteration in gene expression	Molecular function	Biological process	Fold-change *vs* control
1	**PCP2**	Purkinje Cell Protein 2	↑	GTPase regulator activity	GPCR downstream signalling	1.84
2	**PRX**	periaxin	↑	scaffolding protein	axon ensheathment	1.59
3	**RTL1**	Retrotransposon Gag Like 1	↑	Unknown	None by GO term	1.59
4	**CHST14**	Carbohydrate Sulfotransferase 14	↑	sulfotransferase activity	carbohydrate metabolic process	1.40
5	**CYP39A1**	Cytochrome P450 Family 39 Subfamily A Member 1	↑	cytochrome P450 monooxygenase	lipid metabolic process	1.38
6	**CCDC27**	Coiled-Coil Domain Containing 27	↑	unknown	None by GO term	1.35
7	**ARHGEF33**	Rho Guanine Nucleotide Exchange Factor 33	↑	guanine-nucleotide releasing factor	None by GO term	1.32
8	**SSC4D**	Scavenger Receptor Cysteine Rich Family Member With 4	↑	scavenger receptor activity	None by GO term	1.30
9	**MYBPC2**	Myosin Binding Protein C2	↑	actin binding	cell adhesion	1.30

10	**ATOH8**	Atonal BHLH Transcription Factor 8	↑	DNA-binding transcription factor activity	positive regulation of transcription by RNA polymerase II	1.30
11	**EIF4EBP3**	Eukaryotic Translation Initiation Factor 4E Binding Protein 3	↑	translational repressor	negative regulation of translational initiation	1.29
12	**IKZF1**	IKAROS Family Zinc Finger 1	↑	transcription regulator	Regulates transcription through association with both HDAC-dependent and HDAC-independent complexes	1.27
13	**CYREN**	Cell Cycle Regulator Of NHEJ	↑	Protein binding	Negative regulator of DNA repair pathway	1.26
14	**NPR1**	Natriuretic Peptide Receptor 1	↑	Receptor for the brain natriuretic peptide NPPB/BNP	G protein-coupled receptor signaling pathway	1.24
15	**PLA2G4A**	Phospholipase A2 Group IVA	↑	phospholipase A2 activity	phospholipid metabolic process	1.24
16	**CENPO**	Centromere Protein O	↓	component of the interphase centromere complex	centromere complex assembly	-1.09
17	**MYBL1**	MYB Proto-Oncogene Like 1	↓	Transcription factor	positive regulation of transcription	-1.09
18	**KCP**	Kielin Cysteine Rich BMP Regulator	↓	Protein binding	positive regulation of BMP signaling pathway	-1.13
19	**NUDT7**	Nudix Hydrolase 7	↓	fatty acyl-coenzyme A (CoA) diphosphatase	nucleoside diphosphate metabolic process and fatty acid metabolism	-1.17
20	**CHST5**	Carbohydrate Sulfotransferase	↓	sulfotransferase activity	carbohydrate metabolic process	-1.19
21	**TIRAP**	TIR Domain Containing Adaptor Protein	↓	Protein binding	regulation of stress-activated MAPK cascade	-1.21

					positive regulation of IL-6,8,12,15 and TNF production	
22	**CHRNA5**	Cholinergic Receptor Nicotinic Alpha 5 Subunit	↓	cholinergic receptor	Acetylcholine binding and downstream events	-1.24
23	**PDGFC**	Spinal Cord-Derived Growth Factor	↓	Growth factor	positive regulation of MAP kinase activity<br>positive regulation of ERK1 and ERK2 cascade	-1.25
24	**KCTD14**	Potassium Channel Tetramerization Domain Containing 14	↓	Protein binding	Activation of cAMP-Dependent PKA	-1.27
25	**FAM46A**	Terminal Nucleotidyltransferase 5A	↓	Cytoplasmic non-canonical poly(A) RNA polymerase	mRNA stabilization	-1.32
26	**ODF3B**	ciliary microtubule associated protein 1B	↓	Unknown	None by GO term	-1.34
27	**NMRK1**	Nicotinamide Riboside Kinase 1	↓	nucleotide binding	NAD biosynthetic process	-1.34
28	**ITPRIPL2**	Inositol 1,4,5-Trisphosphate Receptor Interacting Protein Like 2	↓	integrant membrane protein	None by GO term	-1.40
29	**6820408C15RIK**	RIKEN cDNA 6820408C15 gene	↓	unknown	None by GO term	-1.46
30	**PRPH**	Peripherin	↓	Class-III neuronal intermediate filament protein<br>structural molecule	Cytoskeleton remodeling	-1.53

Supplementary Table S3.

List of the top 15 genes upregulated or downregulated similarly in the S1 cortex by both burn injury (BI) and formalin-induced inflammatory pain (FA)

	Gene symbol	Gene name	Alteration in gene expression	Molecular function	Biological process	Fold-change *vs* control*
1	**GM21887**	predicted gene, 21877	↑	Unknown	None by GO term	2.82 and 1.59
2	**MT-ATP8**	Mitochondrially Encoded ATP Synthase 8	↑	ATP hydrolysis activity and proton transmembrane transporter activity	ATP biosynthetic process	2.53 and 2.61
3	**SLFN2**	schlafen 2	↑	RNA nuclease activity	rRNA catabolic process apoptotic signaling pathway	2.28 and 1.78
4	**MT-CO2**	Cytochrome C Oxidase Subunit 2	↑	Component of the cytochrome c oxidase	ATP synthesis coupled electron transport	2.15 and 2.11
5	**ISG15**	ISG15 Ubiquitin Like Modifier	↑	ubiquitin protein ligase binding	integrin-mediated signaling pathway	2.10 and 1.99
6	**HBA-A2**	Hemoglobin Subunit Alpha 2	↑	oxygen carrier activity	Cellular responses to stress	2.07 and 1.88
7	**CTLA2A**	cytotoxic T lymphocyte-associated protein 2 alpha	↑	unknown	Immune system process	1.83 and 1.39
8	**ZFP459**	zinc finger protein 459	↑	transcriptional regulator	DNA-templated transcription	1.82 and 1.12
9	**FAM227B**	Family With Sequence Similarity 227 Member B	↑	unknown	None by GO term	1.78 and 1.14
10	**ZFP764**	zinc finger protein 764	↑	DNA-binding transcription factor activity	negative regulation of transcription	1.61 and 1.38

11	**PRSS41**	Serine Protease 41	↑	serine-type endopeptidase activity	proteolysis	1.51 and 1.17
12	**C1QTNF7**	C1q And TNF Related 7	↑	protein binding	None by GO term	1.49 and 1.24
13	**GM10036**	predicted gene 10036	↑	unknown	None by GO term	1.49 and 1.42
14	**MMS22L**	MMS22 Like, DNA Repair Protein	↑	DNA binding protein	DNA repair and DNA damage response	1.48 and 1.04
15	**E130114P18Rik**	RIKEN cDNA E130114P18 gene	↑	unknown	None by GO term	1.42 and 1.03
16	**NKD2**	NKD Inhibitor Of WNT Signaling Pathway 2	↓	protein binding	positive regulation of protein processing	-1.01 and -1.03
17	**HTR2C**	5-Hydroxytryptamine Receptor 2C	↓	G-protein coupled receptor for 5-hydroxytryptamine (serotonin)	G protein-coupled serotonin receptor signaling pathway<br>positive regulation of ERK1 and ERK2 cascade	-1.11 and -1.29
18	**KCNJ2**	Inward Rectifier Potassium Channel 2	↓	inward rectifier potassium channel activity	intracellular potassium ion homeostasis and potassium ion transport	-1.16 and -1.13
19	**P2RX3**	Purinergic Receptor P2X 3	↓	ATP-gated monoatomic cation channel activity	purinergic nucleotide receptor signaling pathway	-1.25 and -1.27
20	**ZFP185**	Zinc Finger Protein 185 With LIM Domain	↓	protein binding	cellular proliferation and/or differentiation	-1.28 and -1.11
21	**FOXP2**	Forkhead Box B2	↓	Transcription factor	regulation of transcription	-1.33 and -1.20

22	**KIRREL**	Kirre Like Nephrin Family Adhesion Molecule	↓	cell adhesion molecule binding	cell-cell adhesion	-1.37 and -1.17
23	**ZFP41**	ZFP41 Zinc Finger Protein	↓	DNA-binding regulatory protein	regulation of transcription	-1.51 and -1.34
24	**BDKRB2**	Bradykinin Receptor B2	↓	receptor for bradykinin	GPCR downstream signalling	-1.75 and -1.65
25	**GM6356**	predicted gene 6356	↓	Unknown	None by GO term	-1.77 and -2.01
26	**TEAD1**	TEA Domain Transcription Factor 1	↓	Transcription enhancer	regulation of transcription	-1.81 and -1.25
27	**ZBTB39**	Zinc Finger and BTB Domain Containing 39	↓	Transcription factor	Negative regulation of transcription	-1.86 and -1.19
28	**THBS4**	Thrombospondin 4	↓	Adhesive glycoprotein	regulation of tissue remodeling	-1.87 and -1.40
29	**GLB1L**	Galactosidase Beta 1 Like	↓	beta-galactosidase and hydrolase activity	carbohydrate metabolic process GAG metabolism	-2.19 and -2.21
30	**PRSS56**	Serine Protease 56	↓	serine-type endopeptidase activity	proteolysis	-2.31 and -1.78

*Respective values in BI and FA.

Supplementary Table S4.

A list of BI-associated Gene Ontology (GO) terms and KEGG pathways in Figure 3a, along with comprehensive bioinformatics data, including false discovery rate (FDR), p-values, and the number of involved genes, as well as other relevant details.

category	term name	description	FDR value	p-value	# background genes	cluster size	# genes	#up-regulated genes	#down-regulated genes
GO Biological Process	GO:0046034	ATP metabolic process	9,6E-03	2,7E-04	165	4562	60	48	12
	GO:0010467	Gene expression	5,0E-08	3,4E-10	2145	4562	592	369	223
	GO:0007269	Neurotransmitter secretion	3,1E-02	1,1E-03	90	4562	36	3	33
	GO:0061001	Regulation of dendritic spine morphogenesis	1,4E-02	4,1E-04	58	4562	28	2	26
	GO:0060998	Regulation of dendritic spine development	9,8E-03	2,8E-04	85	4562	37	4	33
	GO:0010976	Positive regulation of neuron projection development	7,4E-03	2,0E-04	229	4562	78	13	65
	GO:0016570	Histone modification	5,3E-03	1,4E-04	395	4562	122	28	94
	GO:0008038	Neuron recognition	5,2E-03	1,3E-04	53	4562	28	5	23
	GO:1901214	Regulation of neuron death	5,0E-03	1,3E-04	402	4562	124	38	86
	GO:0007416	Synapse assembly	4,8E-03	1,2E-04	97	4562	42	2	40
	GO:0016571	Histone methylation	2,0E-03	4,5E-05	108	4562	47	14	33
	GO:0046928	Regulation of neurotransmitter secretion	4,2E-05	5,4E-07	130	4562	61	6	55
	GO:0042391	Regulation of membrane potential	4,2E-05	5,4E-07	487	4562	161	31	130
	GO:0016358	Dendrite development	6,1E-06	6,4E-08	148	4562	70	5	65
	GO:0007268	Chemical synaptic transmission	1,2E-06	1,1E-08	379	4562	141	16	125
	GO:0048167	Regulation of synaptic plasticity	2,1E-07	1,7E-09	239	4562	104	11	93
	GO:0007267	Cell-cell signaling	1,3E-08	7,8E-11	909	4562	294	46	248
	GO:0061564	Axon development	1,1E-08	6,9E-11	431	4562	166	17	149
	GO:0099177	Regulation of trans-synaptic signaling	5,5E-11	2,1E-13	542	4562	209	24	185
	GO:0044237	Cellular metabolic process	2,0E-22	7,7E-26	6530	4562	1729	782	947

category	term name	description	FDR value	p-value	# background genes	cluster size	# genes	#up-regulated genes	#down-regulated genes
GO Cellular Component	GO:0005753	Mitochondrial proton-transporting ATP synthase complex	1,4E-02	9,0E-04	20	4562	14	14	0
	GO:0005761	Mitochondrial ribosome	2,4E-03	1,3E-04	94	4562	41	40	1
	GO:0005739	Mitochondrion	1,4E-04	5,5E-06	1956	4562	506	323	183
	GO:0044391	Ribosomal subunit	2,2E-15	2,6E-17	207	4562	120	117	3
	GO:0060077	Inhibitory synapse	4,8E-02	4,2E-03	28	4562	15	0	15
	GO:0005925	Focal adhesion	3,0E-02	2,4E-03	196	4562	63	4	59
	GO:0099091	Postsynaptic specialization, intracellular component	2,1E-02	1,5E-03	39	4562	20	1	19
	GO:0098831	Presynaptic active zone cytoplasmic component	1,8E-02	1,3E-03	21	4562	14	0	14
	GO:0030863	Cortical cytoskeleton	1,8E-02	1,2E-03	119	4562	44	7	37
	GO:0098839	Postsynaptic density membrane	1,5E-02	1,0E-03	114	4562	43	2	41
	GO:0032589	Neuron projection membrane	1,5E-02	1,0E-03	79	4562	33	3	30
	GO:0031045	Dense core granule	4,1E-03	2,5E-04	35	4562	21	6	15
	GO:0048786	Presynaptic active zone	3,2E-03	1,9E-04	103	4562	43	4	39
	GO:0043198	Dendritic shaft	2,7E-03	1,5E-04	69	4562	33	1	32
	GO:0098982	GABA-ergic synapse	2,1E-03	1,1E-04	110	4562	46	3	43
	GO:0043194	Axon initial segment	2,1E-03	1,1E-04	27	4562	19	1	18
	GO:0012506	Vesicle membrane	7,2E-04	3,5E-05	935	4562	259	58	201
	GO:0060076	Excitatory synapse	6,1E-04	2,9E-05	80	4562	39	0	39
	GO:0044306	Neuron projection terminus	6,1E-04	2,9E-05	220	4562	80	11	69
	GO:0044304	Main axon	2,2E-04	9,1E-06	84	4562	42	4	38
	GO:0099240	Intrinsic component of synaptic membrane	7,0E-05	2,6E-06	252	4562	94	1	93
	GO:0015629	Actin cytoskeleton	2,3E-05	7,9E-07	510	4562	166	36	130
	GO:0097708	Intracellular vesicle	2,0E-05	6,6E-07	2150	4562	561	142	419
	GO:0150034	Distal axon	1,9E-05	6,1E-07	420	4562	143	17	126
	GO:0042734	Presynaptic membrane	4,7E-07	1,3E-08	214	4562	93	3	90
	GO:0044309	Neuron spine	2,2E-07	5,8E-09	236	4562	101	12	89

category	term name	description	FDR value	p-value	# background genes	cluster size	# genes	#up-regulated genes	#down-regulated genes
GO Cellular Component	GO:0098796	Membrane protein complex	3,0E-09	6,4E-11	1230	4562	376	148	228
	GO:0098978	Glutamatergic synapse	2,6E-09	5,3E-11	522	4562	192	14	178
	GO:0098793	Presynapse	2,4E-09	4,8E-11	687	4562	237	28	209
	GO:0097060	Synaptic membrane	2,1E-11	3,6E-13	479	4562	190	13	177
	GO:0030424	Axon	8,1E-13	1,3E-14	847	4562	299	47	252
	GO:0036477	Somatodendritic compartment	5,1E-13	7,8E-15	1168	4562	385	63	322
	GO:0098794	Postsynapse	4,0E-19	2,5E-21	801	4562	318	47	271
GO Molecular Function	GO:0019843	rRNA binding	2,1E-02	2,7E-04	75	4562	34	32	2
	GO:0022804	Active transmembrane transporter activity	4,2E-02	6,8E-04	399	4562	118	41	77
	GO:0005216	Ion channel activity	2,3E-02	3,1E-04	441	4562	131	23	108
	GO:0022843	Voltage-gated cation channel activity	1,6E-02	1,9E-04	148	4562	56	3	53
	GO:0005509	Calcium ion binding	1,8E-03	1,3E-05	722	4562	211	51	160
	GO:0005215	Transporter activity	7,1E-04	4,2E-06	1172	4562	324	89	235
	GO:0003779	Actin binding	2,4E-04	1,0E-06	459	4562	152	24	128
	GO:0031267	Small GTPase binding	1,3E-05	3,3E-08	288	4562	113	15	98
	GO:0008092	Cytoskeletal protein binding	4,4E-08	7,1E-11	1033	4562	326	67	259
	GO:0043167	Ion binding	6,5E-15	4,0E-18	5972	4562	1540	439	1101
KEGG Pathways	mmu04723	Retrograde endocannabinoid signaling	3,5E-04	1,3E-05	144	4562	60	31	29
	mmu03010	Ribosome	2,2E-14	6,7E-17	126	4562	89	89	0
	mmu04512	ECM-receptor interaction	4,6E-02	8,0E-03	86	4562	31	2	29
	mmu04728	Dopaminergic synapse	4,6E-02	7,9E-03	127	4562	42	9	33
	mmu04810	Regulation of actin cytoskeleton	4,2E-02	6,6E-03	211	4562	64	6	58
	mmu04140	Autophagy - animal	4,0E-02	6,1E-03	136	4562	45	7	38
	mmu04730	Long-term depression	4,0E-02	5,9E-03	59	4562	24	3	21
	mmu04727	GABAergic synapse	4,0E-02	5,7E-03	87	4562	32	6	26

category	term name	description	FDR value	p-value	# background genes	cluster size	# genes	#up-regulated genes	#down-regulated genes
KEGG Pathways	mmu04722	Neurotrophin signaling pathway	3,3E-02	4,2E-03	114	4562	40	9	31
	mmu04350	TGF-beta signaling pathway	2,8E-02	3,4E-03	94	4562	35	12	23
	mmu04150	mTOR signaling pathway	2,7E-02	3,0E-03	156	4562	52	10	42
	mmu04014	Ras signaling pathway	2,7E-02	2,8E-03	225	4562	70	11	59
	mmu04151	PI3K-Akt signaling pathway	2,7E-02	2,7E-03	353	4562	102	21	81
	mmu04540	Gap junction	2,2E-02	2,2E-03	84	4562	33	3	30
	mmu00512	Mucin type O-glycan biosynthesis	2,1E-02	1,9E-03	28	4562	16	2	14
	mmu04020	Calcium signaling pathway	1,2E-02	9,8E-04	191	4562	64	10	54
	mmu04012	ErbB signaling pathway	9,7E-03	7,9E-04	81	4562	34	3	31
	mmu04724	Glutamatergic synapse	8,6E-03	6,7E-04	111	4562	43	5	38
	mmu04010	MAPK signaling pathway	8,6E-03	6,5E-04	287	4562	90	14	76
	mmu04725	Cholinergic synapse	8,2E-03	5,8E-04	110	4562	43	7	36
	mmu04070	Phosphatidylinositol signaling system	6,2E-03	4,1E-04	94	4562	39	1	38
	mmu04919	Thyroid hormone signaling pathway	4,7E-03	2,8E-04	119	4562	47	10	37
	mmu04072	Phospholipase D signaling pathway	4,7E-03	2,7E-04	147	4562	55	6	49
	mmu04015	Rap1 signaling pathway	4,5E-03	2,3E-04	208	4562	72	10	62
	mmu04510	Focal adhesion	3,0E-03	1,3E-04	196	4562	70	6	64
	mmu04360	Axon guidance	1,5E-04	4,4E-06	175	4562	71	3	68

Supplementary Table S5.

A list of FA-associated Gene Ontology (GO) terms and KEGG pathways in Figure 3b, along with comprehensive bioinformatics data, including the false discovery rate (FDR), p-values, and the number of involved genes, among other details.

category	term name	description	FDR value	p-value	# background genes	cluster size	# genes	#up-regulated genes	#down-regulated genes
GO Biological Process	GO:0048583	Regulation of response to stimulus	3,4E-04	2,1E-08	4029	969	250	130	120
	GO:0006950	Response to stress	1,3E-03	2,4E-07	3453	969	215	125	90
	GO:0030198	Extracellular matrix organization	7,1E-03	4,5E-06	281	969	32	21	11
	GO:0009605	Response to external stimulus	1,1E-02	1,4E-05	2575	969	160	95	65
	GO:0048729	Tissue morphogenesis	1,3E-02	1,8E-05	662	969	55	31	24
	GO:0016055	Wnt signaling pathway	1,9E-02	3,5E-05	271	969	29	13	16
	GO:0001818	Negative regulation of cytokine production	1,9E-02	3,7E-05	301	969	31	19	12
	GO:0034145	Positive regulation of toll-like receptor 4 signaling pathway	3,7E-02	9,8E-05	13	969	6	4	2
	GO:0032642	Regulation of chemokine production	3,7E-02	9,8E-05	111	969	16	10	6
GO Cellular Component	GO:0009986	Cell surface	1,4E-13	7,0E-17	1070	22	16	12	4
	GO:0031225	Anchored component of membrane	2,3E-05	4,5E-08	202	22	6	5	1
	GO:0031012	Extracellular matrix	1,4E-04	6,9E-08	528	969	54	35	19
	GO:0030532	Small nuclear ribonucleoprotein complex	1,5E-04	7,5E-08	70	62	6	4	2
	GO:0098644	Complex of collagen trimers	4,8E-04	3,3E-06	20	28	3	3	0
	GO:0061702	Inflammasome complex	1,7E-03	8,5E-07	21	14	4	3	1
	GO:0043231	Intracellular membrane-bounded organelle	2,0E-03	2,1E-05	11954	80	62	36	26
	GO:0000785	Chromatin	5,3E-03	5,7E-05	706	80	11	6	5
	GO:0005751	Mitochondrial respiratory chain complex IV	2,3E-02	6,7E-05	25	62	3	3	0
	GO:0000228	Nuclear chromosome	2,6E-02	3,1E-04	346	80	7	4	3
	GO:0032993	protein-DNA complex	2,8E-02	3,3E-04	248	80	6	6	0

category	term name	description	FDR value	p-value	# background genes	cluster size	# genes	#up-regulated genes	#down-regulated genes
	GO:0005604	Basement membrane	3,7E-02	5,3E-04	121	28	3	3	0
	GO:0071944	Cell periphery	3,9E-02	2,1E-04	7050	62	34	20	14
	GO:0071133	alpha9-beta1 integrin-ADAM8 complex	4,0E-02	3,7E-05	2	55	2	2	0
	GO:0015630	Microtubule cytoskeleton	7,3E-05	5,3E-07	1390	80	19	9	10
	GO:0099080	Supramolecular complex	4,4E-02	6,4E-04	1421	80	14	2	12
	GO:0031514	Motile cilium	4,4E-02	6,5E-04	282	80	6	2	4
GO Molecular Function	GO:0003677	DNA binding	1,8E-03	3,7E-07	2457	80	26	16	10
	GO:0046914	Transition metal ion binding	2,8E-03	2,8E-06	1089	26	9	5	4
	GO:0019956	Chemokine binding	7,1E-03	7,3E-06	35	22	3	3	0
	GO:0032553	Ribonucleotide binding	1,6E-02	4,5E-05	1947	26	10	7	3
	GO:0016763	Pentosyltransferase activity	1,4E-02	3,8E-05	52	26	3	1	2
	GO:0008509	Anion transmembrane transporter activity	3,2E-02	6,6E-06	318	136	11	5	6
KEGG Pathways	mmu00230	Purine metabolism	1,6E-13	4,7E-16	132	26	10	7	3
	mmu04974	Protein digestion and absorption	4,0E-10	1,2E-12	106	28	8	7	1
	mmu04512	ECM-receptor interaction	1,1E-06	3,4E-09	86	55	7	5	2
	mmu00910	Nitrogen metabolism	3,6E-03	2,2E-05	17	60	3	2	1
	mmu00670	One carbon pool by folate	3,6E-03	2,9E-05	19	60	3	3	0
	mmu00650	Butanoate metabolism	6,9E-03	8,3E-05	28	60	3	3	0
	mmu00260	Glycine, serine and threonine metabolism	1,3E-02	1,9E-04	38	60	3	3	0
	mmu03410	Base excision repair	1,7E-02	1,0E-04	33	55	3	2	1
	mmu00480	Glutathione metabolism	2,3E-02	6,9E-05	71	64	4	3	1
	mmu04110	Cell cycle	3,3E-02	9,9E-05	121	80	5	5	0
	mmu04350	TGF-beta signaling pathway	7,6E-11	2,3E-13	94	15	7	3	4
	mmu00760	Nicotinate and nicotinamide metabolism	1,5E-09	1,4E-11	40	26	6	2	4
	mmu00240	Pyrimidine metabolism	6,9E-07	8,3E-09	55	26	5	2	3

category	term name	description	FDR value	p-value	# background genes	cluster size	# genes	#up-regulated genes	#down-regulated genes
KEGG Pathways	mmu00562	Inositol phosphate metabolism	2,1E-04	6,3E-07	72	20	4	1	3
	mmu04014	Ras signaling pathway	8,2E-04	4,1E-06	225	62	7	2	5
	mmu00740	Riboflavin metabolism	2,4E-03	4,9E-05	7	26	2	0	2
	mmu00071	Fatty acid degradation	2,3E-02	4,2E-04	50	60	3	1	2

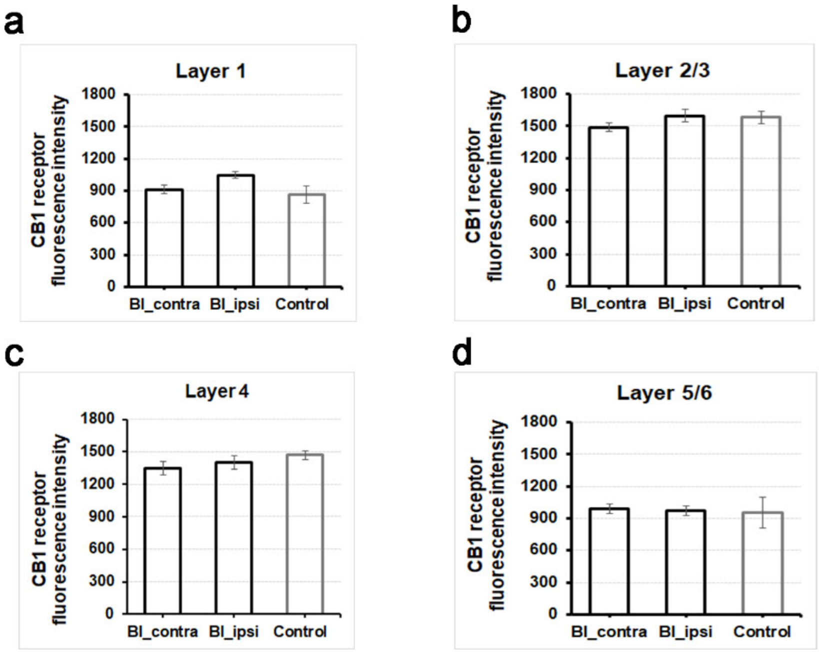

Supplementary Figure S1. Layer-specific CB1 Receptor fluorescence intensity in the S1 cortex shows no significant change one-hour post-burn injury. (**a**) Quantitative analysis of CB1 receptor fluorescence intensity in layer 1 of the S1 cortex, compared to the control. (**b**) Quantitative analysis of CB1 receptor fluorescence intensity in layer 2/3 of the S1 cortex compared to the control. (**c**) Quantitative analysis of CB1 receptor fluorescence intensity in layer 4 of the S1 cortex compared to the control. (**d**) Quantitative analysis of CB1 receptor fluorescence intensity in layer 5/6 of the S1 cortex compared to the control. The data are presented as mean ± SEM (n = 5-8 sections from 2 animals per group). The term BI_contra specifically refers to the right hemisphere of the brain, which lies opposite the site of the burn injury, while BI_ipsi signifies the left-sided cortical region.
